# Toll-like receptor activation induces airway obstruction and hyperresponsiveness in guinea pigs

**DOI:** 10.1186/s12931-024-03050-3

**Published:** 2024-11-29

**Authors:** Yujiao Xiang, Jielu Liu, Mu Nie, Gunnar Nilsson, Jesper Säfholm, Mikael Adner

**Affiliations:** 1 Experimental Asthma and Allergy Research Unit, Institute of Environmental Medicine, Karolinska Institutet, Stockholm, Sweden; 2 Division of Immunology and Allergy, Department of Medicine Solna, Karolinska Institutet, Center for Molecular Medicine, Karolinska University Hospital, Stockholm, Sweden; 3 Department of Medical Sciences, Uppsala University, Uppsala, Sweden; 4 Unit of Integrative Metabolomics, Institute of Environmental Medicine, Karolinska Institutet, Stockholm, Sweden; 5 Department of Respiratory Medicine and Allergy, Karolinska University Hospital, Stockholm, Sweden; 6 Institute of Environmental Medicine, Karolinska Institutet, Nobels väg 13, Stockholm, SE-171 77 Sweden

**Keywords:** Respiratory infections, Poly (I:C), Lipopolysaccharide (LPS), Imiquimod, Inflammation, Mast cell

## Abstract

**Background:**

Microbial infections, particularly those caused by rhinovirus (RV) and respiratory syncytial virus (RSV), are major triggers for asthma exacerbations. These viruses activate toll-like receptors (TLRs), initiating an innate immune response. To better understand microbial-induced asthma exacerbations, animal models that closely mimic human lung characteristics are essential. This study aimed to assess airway responses in guinea pigs exposed to TLR agonists, simulating microbial infections.

**Methods:**

The agonists poly(I: C) (TLR3), lipopolysaccharide (LPS; TLR4) and imiquimod (TLR7), or the combination of poly(I: C) and imiquimod (P/I) were administered intranasally once a day over four consecutive days. The latter group received daily intraperitoneal injections of dexamethasone starting one day before the TLR agonists challenge. Respiratory functions were measured by whole-body plethysmography and forced oscillatory technique. Bronchoalveolar lavage fluid (BALF) cells and lungs were collected for analysis.

**Results:**

The intranasal exposure of LPS and P/I caused an increase in enhanced pause (Penh) after challenge, whereas neither poly(I: C) nor imiquimod alone showed any effect. After the challenges of LPS, poly(I: C) or P/I, but not imiquimod alone, induced an increase of both Rrs (resistance of the respiratory system) and Ers (elastance of the respiratory system). LPS exposure caused an increase of neutrophils in BALF, whereas none of the other exposures affected the composition of cells in BALF. Exposure to LPS, poly (I: C), imiquimod, and P/I all caused a marked infiltration of inflammatory cells and an increase of mast cells around the small airways. For the expression of inflammatory mediators, LPS increased CXCL8, poly(I: C) and imiquimod decreased IL-4 and IL-5, and increased IFNγ. Imiquimod increased CXCL8 and IL-6, whereas P/I decreased IL-5, and increased IL-6 and IFNγ. The increases in Rrs, Ers, and airway inflammation, but not the altered expression of inflammatory cytokines, were attenuated by dexamethasone.

**Conclusions:**

TLR agonists promote acute airway inflammation and induce airway obstruction and hyperresponsiveness in guinea pigs. The severity of these effects varies depending on the specific agonists used. Notably, dexamethasone reversed pulmonary functional changes and mitigated bronchial inflammation caused by the combined treatment of P/I. However, it had no impact on the expression of inflammatory mediators.

**Supplementary Information:**

The online version contains supplementary material available at 10.1186/s12931-024-03050-3.

## Background

Asthma is a heterogeneous disease characterized by airflow obstruction, airway inflammation, airway hyperresponsiveness and airway remodeling. The most common symptoms are cough, wheezing, chest tightness, and breathlessness. Today, asthma affects approximately 300 million people worldwide, resulting in a significant global health care burden [1, 2]. One important cause of airway obstruction in allergic asthma is exposure to allergens, where mast cells become activated and degranulated, releasing contractile and pro-inflammatory mediators such as histamine, proteases, leukotrienes, cytokines, and chemokines, which further lead to inflammation and smooth muscle constriction [3]. Fortunately, with current general asthma management, including inhaled corticosteroids and β_2_-agonists, many patients are well controlled [4]. However, when asthmatic patients suffer from respiratory infections, they may experience exacerbations of their symptoms, necessitating immediate medical attention. The most common cause of asthma exacerbations is viral respiratory infections [5], yet the mechanisms by which these infections lead to airway obstruction remain unclear.

The most frequent respiratory viral infections that trigger asthmatic exacerbations are rhinovirus (RV) and respiratory syncytial virus (RSV) [6]. When microorganisms enter the body, they are recognized by pattern recognition receptors (PRRs) and activate innate immune system as the first line of defense. There are several types of PRRs, of which Toll-like receptors (TLRs) are one family. There are 10 different TLRs in human [7, 8]. Previous studies have shown that when RV, RSV, and SARS-CoV-2 infect epithelium, their single-stranded RNA (ssRNA) genomes can be directly detected by TLR7 in endosomes [9, 10]. Moreover, viruses can replicate in airway epithelial cells, producing double-stranded RNA (dsRNA) that is recognized by TLR3 receptors present on epithelial cells or dendritic cells [11]. Also, gram-negative bacterial infections such as *Moraxella catarrhalis* and *Haemophilus influenzae* account for a large proportion of early clinical respiratory tract infections in children and can cause exacerbations. LPS, a major component of the outer membrane of Gram-negative bacteria, is recognized by TLR4 present on airway epithelial cells [12]. Although it has been shown that activation of both TLR3 and TLR4 can cause an increase of contractile receptors on the airway smooth muscle, TLRs do not cause direct contractions [13]. Instead, it is possible that the inflammatory activation causes a release of contractile substances that induce airway obstruction.

To investigate how bacterial and viral infection causes airway narrowing and exacerbation, a model relevant to small airway contraction and mast cells is needed. Guinea pigs were selected for this study because, compared to mouse model, the response of their airways to acute allergenic stimulation is almost identical to that of humans, and their airway neural control is very similar to humans [14, 15]. In contrast, mouse models have several limitations when studying asthma exacerbation as they are different from humans regarding the pharmacology of their smooth muscle, and they have a limited number of mast cells in the lung [16]. To investigate the effect of microbial infections in guinea pigs, TLR agonists were administered to mimic airway infections. Furthermore, to investigate the acute effects of corticoid treatment on specifically viral stimulation, dexamethasone was given when TLR3 and TLR7 agonists were co-administered.

## Methods

### Animals

Female Dunkin-Hartley guinea pigs weighing between 300 and 400 g were obtained from Envigo (Horst, The Netherlands) and housed in Astrid Fagræus laboratorium (Solna, Sweden) on a 12/12 h light/dark cycle with food and water provided *ad libitum*. The choice of sex was made to prevent physical conflicts in the cages. All experiments were initiated at least one week after the animals had acclimated and were approved by the Stockholm Animal Research Ethics Committee (Permit number: 10973 − 2019 and 21900 − 2022).

### Challenge protocol

After a brief anesthesia with 5% isoflurane, guinea pigs were intranasally challenged every day for four consecutive days with PBS, 0.25 µg/g LPS (L4391, Sigma-Aldrich, Burlington, United States), 2.5 µg/g poly (I: C) (P9582, Sigma-Aldrich), vehicle (PBS containing 25% DMSO), 0.3 µg/g imiquimod (I5159, Sigma-Aldrich), or the combined use of poly(I: C) and imiquimod (P/I). In addition, the animals that were given P/I were also treated with 20 mg/kg dexamethasone (011021, Abboxia, Mölndal, Sweden) [17, 18], one day before the start of the experiment and then one hour before each challenge by intraperitoneal injection.

### Non-invasive measurements of respiratory responses

After each challenge the respiratory patterns of the guinea pigs were recorded by whole-body plethysmograph (EMMS, Hampshire, UK) for one hour. Each guinea pig was placed in a ventilated chamber. Sensors on the top of the chamber detected changes in breathing pattern. Enhanced pause (Penh) was acquired by eDacq Software version 1.8 (EMMS).

### Invasive measurements of lung mechanics

One day after the last challenge, guinea pigs were anesthetized with 40 mg/kg ketamine hydrochloride (MSD Animal Health, Sweden) and 0.75 mg/kg medetomidine hydrochloride (Vetmedic, Sweden). Animals were tracheostomized, intubated, and ventilated using the FlexiVent system (Scireq) with the FX4 module. They were exposed to PBS aerosols for 10 s, followed by baseline measurements of respiratory mechanics, including Rrs and Ers, using a single-compartment model. Increasing concentrations of methacholine (A2251, Sigma-Aldrich) at 0.03125, 0.0625, 0.094, 0.125, 0.19, and 0.25 mg/mL were then administered by nebulization at 7-minute intervals. The blood oxygen saturation levels (SpO₂) and heart rates of the guinea pigs were continuously monitored throughout the procedure. The experiment was terminated when these measurements became undetectable.

### Bronchoalveolar lavage fluid (BALF) cells analysis

Animals underwent lavage with 5 mL of sterile PBS twice. The obtained fluid was centrifuged and the BALF cells were resuspended for counting. Then, 50,000 cells in 100 µl PBS were loaded on cytospin slides and stained with May Grünwald - Giemsa. Differential cell counts were performed on 300 randomly selected cells using a microscope.

### Lung tissue histology

After in vivo experiment, right caudal lung lobe was divided into two equal parts. One segment underwent fixation in Carnoy’s solution (60% ethanol, 30% chloroform and 10% acetic acid), followed by Astra Blue-Hematoxylin staining for mast cells quantification. Simultaneously, the other segment was fixed in 4% buffered formaldehyde for Haematoxylin-Eosin (H&E) staining to analyze airway inflammation. Images of stained slides were taken at × 20 magnification using a Zeiss AxioScan.Z1 slide scanner (ZEISS, Oberkochen, Germany). Mast cells quantification was conducted through manual counting. The ZEN software (version 3.3, blue version, ZEISS) was employed to calculate the inflammatory infiltration area and bronchial diameter. Quantifications were conducted by analyzing all small airways present in the whole lung images in a blinded manner, and the results were normalized to the airway diameter.

### Quantitative real-time polymerase chain reaction (qRT-PCR)

Tissue samples around main airway branches were removed by a circular punch with a diameter of 8 mm, followed by preservation in RNA later (R0901, Sigma-Aldrich). Subsequently, RNeasy Plus Kits (74134, Qiagen, Venlo, Netherlands) were employed to extract tissue RNA and eliminate genomic DNA. The A260/A280 ratio of the RNA samples ranged from 1.9 to 2.1, and the A260/A230 ratio ranged from 2.0 to 2.2. QuantiTect Reverse Transcription Kit (205311, Qiagen) was used for cDNA synthesis. The mRNA level of IL-4, IL-5, IL-6, CXCL8, interferon (IFN)γ and the housekeeping genes β-actin (Table 1) were measured by 7500/7500 Fast Real-Time PCR System (Applied Biosystems, Foster City, United States). Changes in gene expression were determined using comparative delta Ct (△△Ct) method.

**Table 1:** Primer sequences used in qRT-PCR

	Forward	Reverse
IL-4	GCCCAAACAGAGAGGGAGAC	TCACTCACTGGACAGTTCGAC
IL-5	GGGAAGCTCTGGCAACACTA	AACTGCTTCACTCTCCGTCG
IL-6	AAGTTCCTCTCCACAAGCACC	AAGTCGTGCTGAACTTGTGC
CXCL8	GTGACAATCGACAGCTCTGC	CTTGGCTCTCAGTCCTCTTCAA
IFNγ	CAACAAGGTGCAGGCTTTCAA	TCTTCGTTTCCTCTGGTTCGG
β-actin	ATTGCCGACAGGATGCAGAA	CTGCTGGAAGGTGGAGAGTG

### Statistical analysis

All data are presented as mean ± standard error of the mean (SEM) and were analyzed using GraphPad Prism 10.1 (San Diego, California, USA). For experiments involving TLR alone, statistical analysis compared each group to its respective control group. Lung function measurement and differential cell count analyses considered two independent categorical variables: group and one of the following factors: challenge time, Mch concentration, or cell types. A two-way ANOVA with Sidak’s post hoc test was conducted, as the groups were independent, with no overlap between the different TLR agonist administration groups. For PCR data and histological analysis, a single categorical variable (group) was considered, and unpaired t-tests were applied. In the study involving the combined use of P/I, lung function measurement and differential cell count analyses were using two-way ANOVA, with Tukey’s post hoc tests for broad comparisons among groups. Histological and PCR data were analyzed using one-way ANOVA, with Dunnett’s test to compare each group to the P/I combination group without treatment. A p-value of less than 0.05 was considered statistically significant.

## Results

### Selective activation of TLR3 and TLR4, but not TLR7, induces functional changes in the airways

**Fig. 1:**
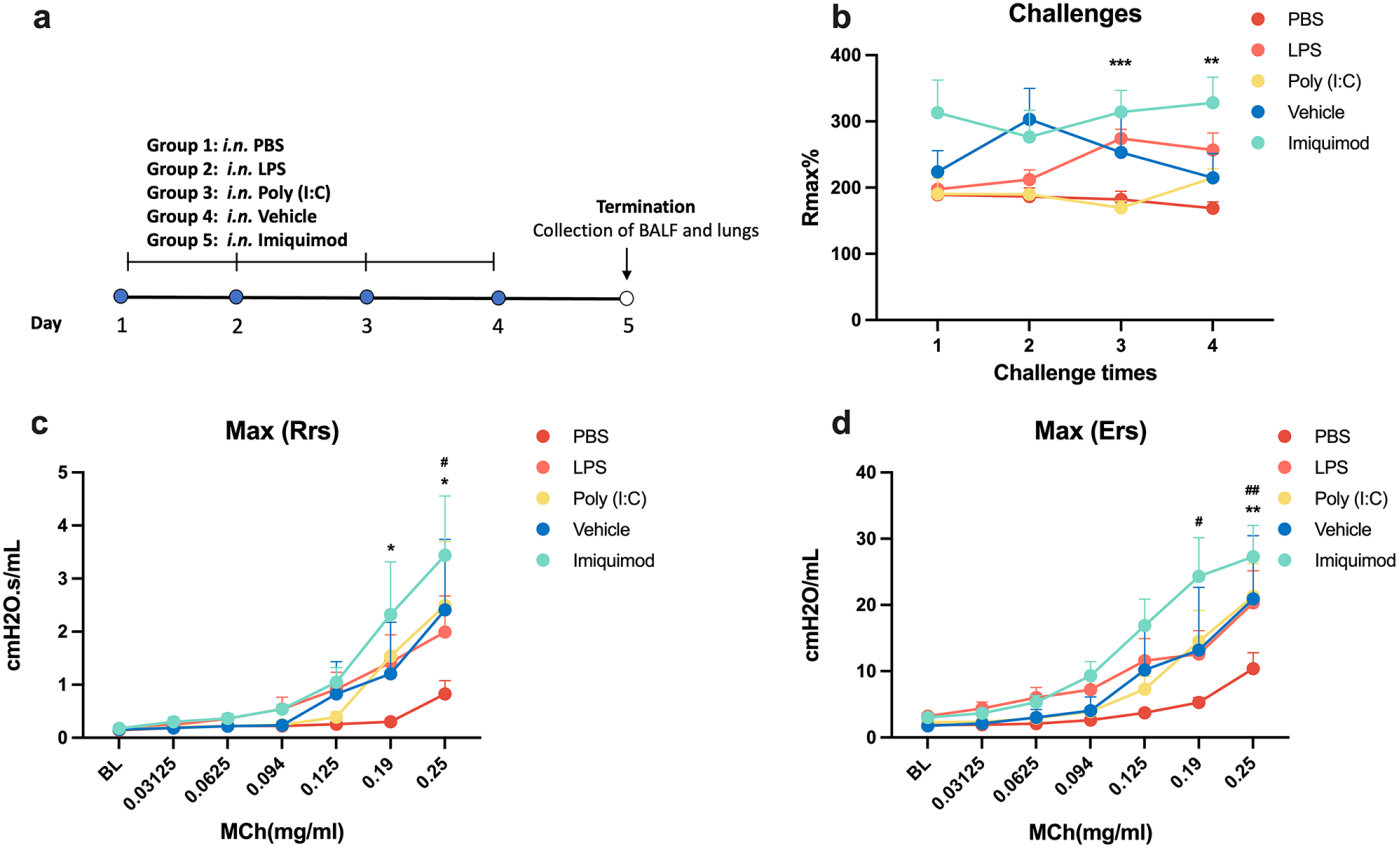
The effects of TLR agonists on airway function. (**a**) Schematic representation of the experimental protocol. The vehicle group included 3 animals, and each of the other groups comprised 8 animals. (**b**) The maximum Penh values (Rmax) of each challenge. Two-way ANOVA with Sidak’s post hoc test: **PBS vs. LPS, *p* < 0.01; ***PBS vs. LPS, *p* < 0.001. (**c**) Differences of resistance of respiratory system (Rrs). ‘BL’: baseline. Two-way ANOVA with Sidak’s post hoc test: ^*^PBS vs. LPS, *p* < 0.05; ^#^PBS vs. Poly(I: C), *p* < 0.05. (**d**) Differences of elastance of the respiratory system (Ers). Two-way ANOVA with Sidak’s post hoc test:^**^PBS vs. LPS, *p* < 0.01; ^#^PBS vs. Poly(I: C), *p* < 0.05; ^##^PBS vs. Poly(I: C), *p* < 0.01

Guinea pigs were given PBS, LPS, poly (I: C), imiquimod or vehicle *i.n.* in a four-day model with concurrent monitoring of body weight (Fig. 1a). The weight curve analysis revealed that animal weights were not significantly different at baseline or during the period of TLR agonist exposure (Supplementary Fig. 1). Analysis of the maximal Penh responses (R_max_) within one hour after each exposure to TLR agonists, showed that neither poly (I: C) nor imiquimod demonstrated any discernible effect on Penh throughout the study. However, following the third and fourth challenge, LPS induced a notable increase in Penh over the baseline value (*P* < 0.05; Fig. 1b), indicating a potential presence of airflow obstruction. The airway responses during the whole time for different agonists were shown in supplementary Fig. 2.

To further study airway function, the response to methacholine was evaluated by flexiVent one day after the fourth challenge. The results revealed that LPS and poly (I: C) elevated Rrs (Fig. 1c) and Ers (Fig. 1d), implying that activation of both TLR3 and TLR4 causes airway hyperresponsiveness (AHR). Although the TLR7 agonist, imiquimod, showed increase effects in Rmax, Rrs and Ers, it did not exert a significant impact on airway functions when compared to effect with DMSO which was used to dissolve imiquimod.

### TLR3, TLR4, and TLR7 stimulation induce airway inflammation and increase mast cell numbers

**Fig. 2:**
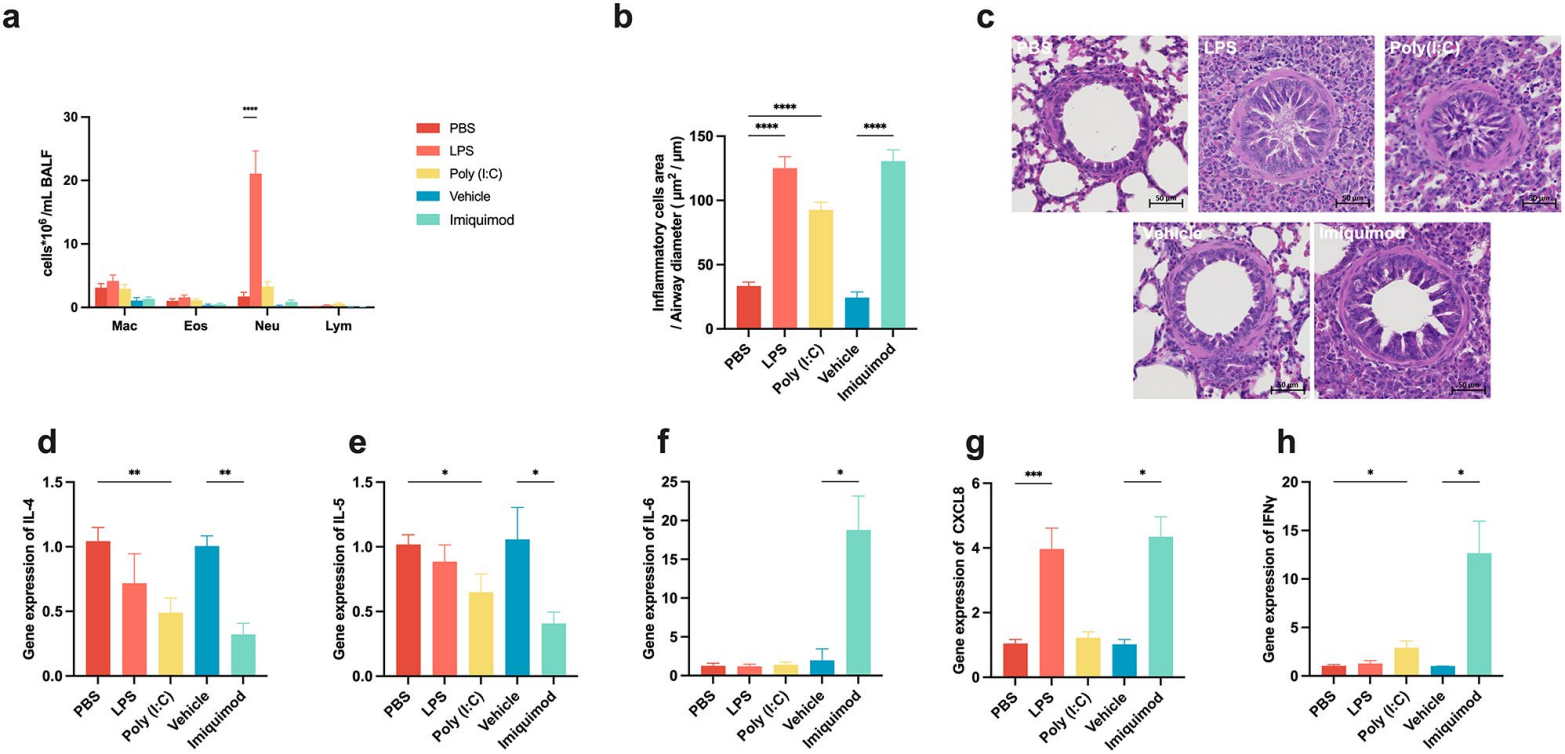
The effects of TLR agonists on inflammatory responses. (**a**) Differential inflammatory cells count in bronchial lavage fluid. Two-way ANOVA with Sidak’s post hoc test: ^****^p < 0.0001. (**b**) Analysis of inflammatory infiltrate area around the airways. Unpaired t-test: ^****^p < 0.0001. (**c**) Representative histological sections of H&E-stained lungs. (d-h) The mRNA expression of IL-4, IL-5, IL-6, CXCL8 and IFNγ in lung tissue. Unpaired t-test: ^*^p < 0.05, ^**^p < 0.01, ^***^p < 0.001

**Fig. 3:**
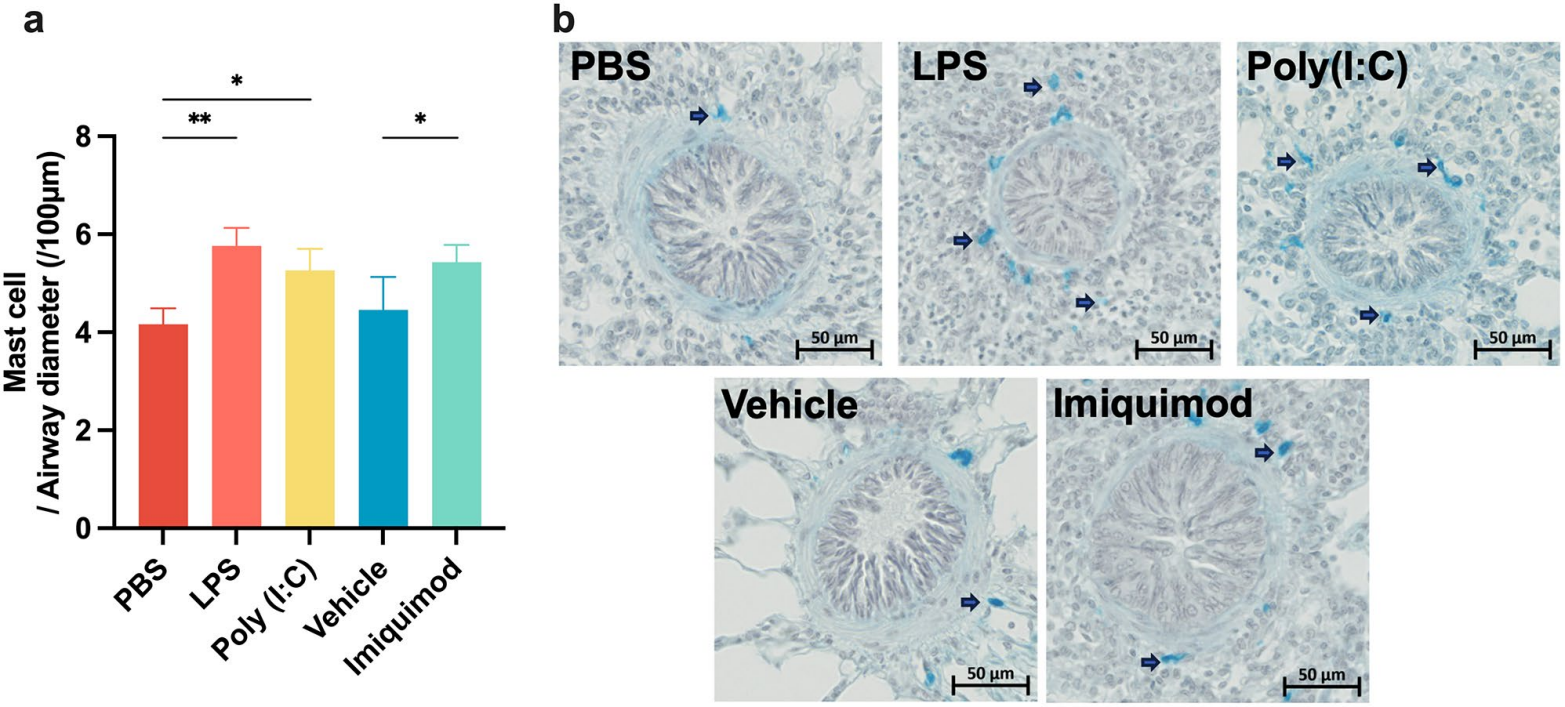
TLR agonists increase the number of mast cells in lung tissues. (**a**) The number of mast cells normalized by airway diameter. Unpaired t-test: ^*^p < 0.05, ^**^p < 0.01. (**b**) Representative histological sections of Astra Blue-Hematoxylin-stained lungs. The blue dots around the airways are mast cells

Analysis of cells in BALF showed that LPS significantly induced an increase of the numbers of neutrophils, whereas the other TLR agonists had no effect on the number of inflammatory cells in BALF (Fig. 2a). In contrast, LPS, poly (I: C) and imiquimod caused a marked inflammatory infiltration in the lung around the small airways (Fig. 2b-c). Transcript levels of selected inflammatory mediators (Fig. 2d-h) indicated that LPS upregulated CXCL8 mRNA levels, while poly (I: C) upregulated IFNγ mRNA levels and down-regulated IL-4 and IL-5 mRNA levels. Imiquimod caused higher IL-6, CXCL8 and IFNγ mRNA expression and reduced IL-4 and IL-5 mRNA expression. Using Astra Blue-Hematoxylin staining to examine mast cell changes in lung tissue showed that all three TLR agonists led to an increase in the number of mast cells (Fig. 3a-b).

### Dexamethasone prevents the TLR3 and TLR7 induced changes in lung function

**Fig. 4:**
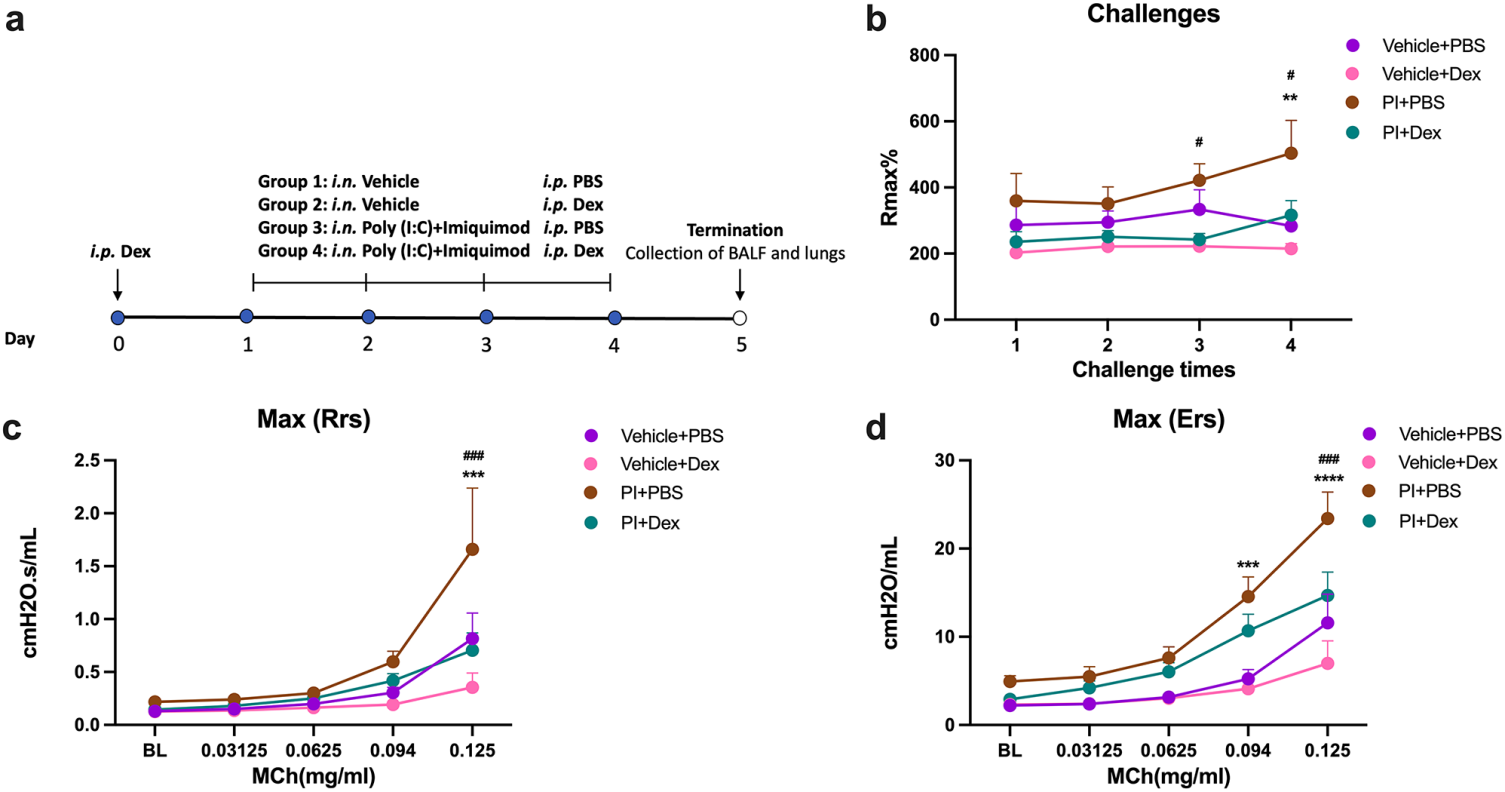
Dexamethasone mitigates Toll-like receptor (TLR) agonist-induced airway functional alterations. (**a**) Schematic illustration of experimental protocol. Each group consists of 8 animals. (**b**) The maximum Penh values (Rmax) of each challenge. (**c**) Differences of resistance of respiratory system (Rrs). (**d**) Differences of elastance of the respiratory system (Ers). Two-way ANOVA, followed by Tukey’s post hoc tests: ^**^Vehicle + PBS vs. P/I + PBS, *p* < 0.01; ^***^Vehicle + PBS vs. P/I + PBS, *p* < 0.001; ^****^Vehicle + PBS vs. P/I + PBS, *p* < 0.0001. ^#^P/I + PBS vs. P/I + Dex, *p* < 0.05; ^###^P/I + PBS vs. P/I + Dex, *p* < 0.001

To simulate combined viral infections, poly (I: C) and imiquimod (P/I) were simultaneously intranasally administered. Additionally, to assess whether P/I was affected by steroids, we treated the guinea pigs with dexamethasone (Fig. 4a). Throughout the entire study, the interventions had no significant impact on the animal’s body weight (Supplementary Fig. 3). Analysis of plethysmograph data demonstrated that P/I induced an increase of Penh after the third and fourth exposure (Fig. 4b). Supplementary Fig. 4 depicts the airway responses over one hour after each challenge. Moreover, P/I increased the methacholine responses of both Rrs (Fig. 4c) and Ers (Fig. 4d). Treatment with dexamethasone one day prior to exposure and one hour before subsequent exposure, reversed the Penh, Rrs and Ers changes caused by P/I (Fig. 4b-d).

### Dexamethasone prevents TLR3 and TLR7 agonists-induced inflammation and mast cells increase in lung tissue

**Fig. 5:**
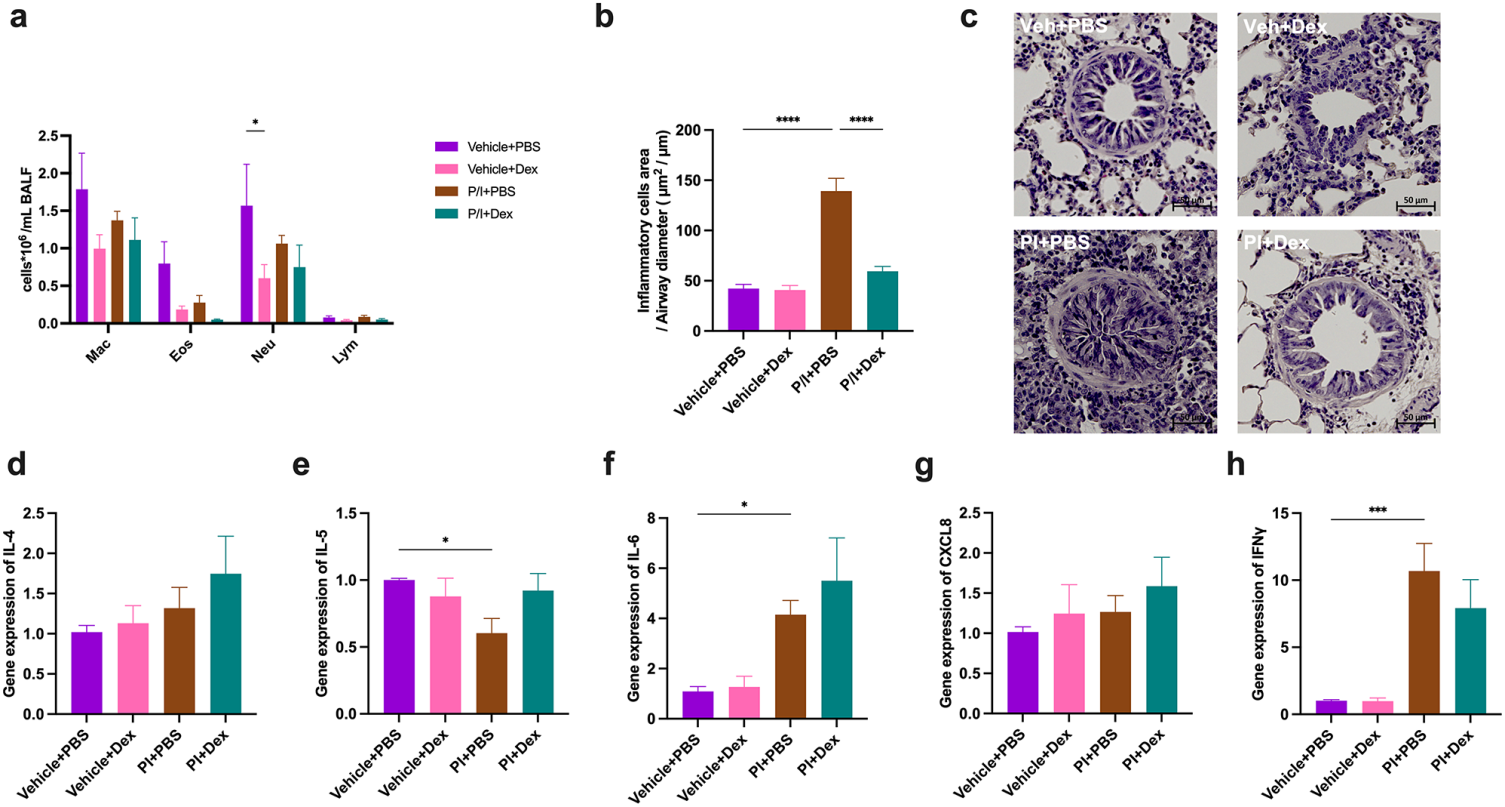
Dexamethasone alleviates Toll-like receptor (TLR) agonist-induced inflammation. (**a**) Inflammatory cells count in Bronchial lavage fluid. Two-way ANOVA, followed by Tukey’s post hoc tests: ^*^p < 0.05. (**b**) Analysis of inflammatory infiltrate area around the small airways. One-way ANOVA, followed by Dunnett’s post hoc tests: ^****^p < 0.0001. (**c**) Representative histological sections of H&E-stained lungs. (d-h) The mRNA expression of IL-4, IL-5, IL-6, CXCL8 and IFNγ in lung tissue. One-way ANOVA, followed by Dunnett’s post hoc tests: ^*^p < 0.05, ^***^p < 0.001

**Fig. 6:**
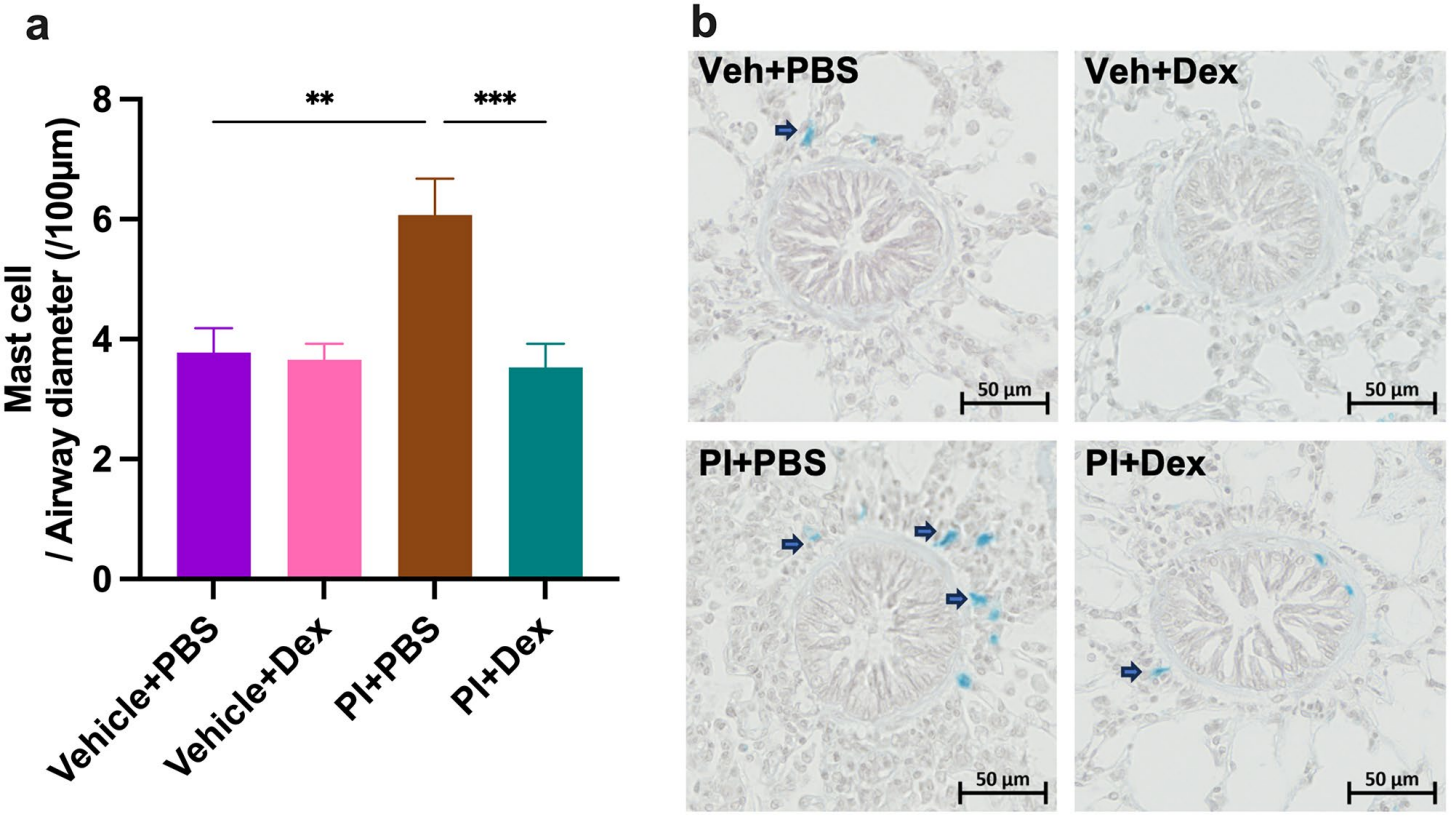
Dexamethasone alleviates the TLR agonist-induced increase in mast cell numbers. (**a**) The number of mast cells. One-way ANOVA, followed by Dunnett’s post hoc tests: ^**^p < 0.01, ^***^p < 0.001. (**b**) Representative histological sections of Astra Blue-Hematoxylin-stained lungs. The blue dots around the airways are mast cells

When examining the effect of P/I on the inflammatory parameters, no significant effect was found on the number of cells in BALF (Fig. 5a), whereas a marked infiltration of cells was found in the lung around the airways (Fig. 5b-c). This infiltration was prevented by the dexamethasone treatment (Fig. 5b-c). PCR analysis showed that P/I significantly up-regulated the mRNA expression of IL-6 and IFNγ, while significantly down-regulating the expression of IL-5. Dexamethasone had no effect on these cytokines (Fig. 5d-h). In addition, the combined treatment with TLR3 and TLR7 caused an augment in the number of mast cells, which did not appear by the treatment of dexamethasone (Fig. 6a-b).

## Discussion

In the present study, guinea pigs were exposed intranasally to TLR agonists over four days to investigate the airway responses to a simulated microbial infection. The results showed that both LPS and P/I significantly induced airway obstruction after the third and fourth challenge. Furthermore, the measurement of airway responsiveness showed that LPS, poly (I: C) and P/I induced AHR by increasing both Rrs and Ers. All TLR exposures caused a marked airway inflammation and increase in mast cell numbers around the airways, whereas only LPS caused an infiltration of neutrophils in the BALF. The different TLR stimulations induced changes in the expression of different inflammatory mediators. The treatment with dexamethasone reversed the alterations induced by P/I, except for the inflammatory cytokine expression profile.

Both viral and bacterial respiratory infections cause wheezing due to bronchoconstriction [19, 20]. To address the potential of TLR agonists to cause decreased lung function, the stimuli were administered over four days to mimic an airway infection. The direct responses were measured by Penh, which records the breathing pattern influenced by several different sources such as swollen airways and release of contractile substances [21]. In these experiments, stimulation with LPS and P/I resulted in an increased Penh response after challenge, while poly (I: C) and imiquimod alone did not induce increased Penh. An increase in direct response to LPS has been shown before in guinea pigs [22]. Although it has been shown that activation of TLR3, TLR4 and TLR7 activate cultured smooth muscle cells [23–25], it has as far as we know not been shown that direct activation of any of these TLRs cause contraction of airway smooth muscle. Instead, relaxations in guinea pig trachea and inhibition of contractions have been shown to be caused by several TLR7 agonists [26, 27]. However, this has been shown with high concentrations and through a TLR7-independent pathway due to its quinoline properties [28], and any signs of this were not seen in our study. The marked increase of the Penh response when poly(I: C) and imiquimod were used together, suggests a potential interaction between the TLR3 and TLR7 pathways. This interaction may arise because these two TLR subtypes activate different intracellular pathways—myeloid differentiating factor 88 (MyD88) for TLR7 and Toll/IL-1R domain-containing adaptor-inducing IFN-β (TRIF) for TLR3. In contrast, TLR4 can activate both pathways, which also contributed to the increased Penh response [29]. However, further investigations are needed to confirm this interaction.

The measurement of airway responsiveness to methacholine one day after the last challenge showed that stimulation by LPS, Poly(I: C) and P/I caused increased lung resistance and elastance, indicating that at least stimulation of TLR3 and TLR4 can induce AHR in guinea pigs. These findings are in accordance with previous studies in mice and for TLR4 in guinea pigs [22, 30]. In addition, in vitro studies using isolated mouse trachea have demonstrated that increased contraction to bradykinin is associated with altered smooth muscle properties induced by LPS and poly(I: C) [31, 32]. It has also been shown that TLR3 activation induce the release of mediators such as IL17A and IL-33, subsequently leading to mucous metaplasia and AHR in mice [33, 34]. Stimulating TLR7 has been proposed as a treatment for airway hyperresponsiveness (AHR) induced by allergic airway inflammation, a finding supported by studies in both rats and mice [35, 36]. On the other hand, TLR7 activation has also been implicated in the development of AHR in the context of viral infections, as demonstrated in mice 42 days post-infection [37]. Additionally, human precision-cut lung slices (PCLS) from asthmatic patients have shown enhanced AHR responses to carbachol following infection with RV39 [38]. However, 4 days of TLR7 stimulation alone did not show a significant AHR, which is in accordance with similar time period of stimulation in mice [39]. Nevertheless, the AHR induced by poly (I: C) was still present when given in combination with imiquimod.

The histopathological analysis of the lungs revealed that all used TLR agonists induced a marked inflammation. Only LPS resulted in neutrophilic inflammation in BALF. These findings closely align with earlier studies in mice exposed to poly (I: C) at nearly the same dosages as used in the present study (2 µg/g versus 2.5 µg/g) and to R848, another TLR7 agonist, for 4 days [30, 39, 40]. The increase in neutrophils in BALF following LPS exposure may be attributed to LPS-induced increased membrane permeability and lung injury [41], while poly (I: C) and imiquimod show no significant impact on permeability [42, 43]. Although we found that imiquimod did not cause a significant increase in AHR, TLR agonists-induced lung inflammation may be related to AHR. During RSV infection, activated Th17 lymphocytes enhances the contractile response of mouse and human airway smooth muscle [44, 45], which may influence the degree of AHR [46]. Hence, alterations in inflammatory cells might account for changes in airway function. Another important finding is the increase in lung mast cell numbers after TLR stimulation. Earlier studies demonstrated that SARS CoV-2 induced massive activation of mast cells during infection [47]. Analysis of lung tissue from young children with viral lower respiratory tract infections also revealed that viral infections lead to an increase in mast cells in the alveolar parenchyma [48]. Mast cells could release histamine, inducing airway remodeling and leading to sustained airflow limitation in asthma [49]. As we earlier have shown that a decrease of both inflammation and mast cells by monensin can decrease AHR in a guinea pig asthma model [50], the TLR-induced increase in inflammatory cells and mast cells might represent one mechanism underlying its induction of airway constriction and AHR.

Investigations of the expression of the tissue surrounding the airways revealed that the viral TLR stimulations of poly (I: C) and imiquimod, alone or in combination, generally caused an upregulation of the Th1 and downregulation of the Th2 inflammatory mediators measured. However, as these changes are measured at the transcriptional level only, their specific pathological contribution to pathogenesis are still to be determined. LPS only increased CXCL8 which probably is linked to the neutrophil increase [51]. The combined use of P/I upregulated IL-6 and IFNγ while downregulating IL-5. In vitro, rhinovirus, which activates both TLR3 and TLR7 receptors, could cause the release of proinflammatory mediators IL-6, and CXCL8 from bronchial epithelial cells (BECs) and mast cells [52, 53]. Sequencing of BECs from asthmatic patients during RV infection showed a significantly enhanced IFNγ response [54]. Moreover, patients with severe/critical COVID-19 showed increased levels of Th1 cytokines and decreased levels of Th2 cytokines (IL-5) [55]. In RSV-infected mice, IL-5 expression decreased day by day [56]. Thus, in contrast to the Type 2 (T2 or eosinophilic) type of asthma which was mediated by IL-4, IL-5, and IL-13, the profile of the inflammatory mediators with high levels of IL-6, CXCL8, and IFNγ after TLR stimulation mirrored that of the non-T2 endotype of asthma [57–59]. Furthermore, it is interesting that in this study, both poly(I: C) and imiquimod alone suppressed IL-4 expression, whereas their combination showed no significant effect. This variation of response suggests a crosstalk between TLR signaling pathways. Studies shown that TLR7 promoted IL-10 secretion [60, 61], and the elevated IL-10 levels could inhibit the production of IL-4 [62]. In contrast, TLR3 has shown to suppress IL-10 secretion by upregulating endothelial lipase [63], thereby interfering with the regulatory effect of TLR7 on IL-4. It is possible that a crosstalk between TLRs may account for the inconsistent expression of cytokines observed with imiquimod alone compared to its combined used with poly (I: C), warranting further investigation.

In the present study, the treatment of dexamethasone in P/I-challenged guinea pigs prevented the increase of both Penh and the lung resistance as well as the influx of inflammatory cells and mast cells in the lung. The inhibition by dexamethasone on AHR and neutrophils has previously been shown in guinea pigs exposed to LPS [64]. Dexamethasone has also been shown in mouse models to reduce the number of tissue-resident mast cells by decreasing the secretion of stem cell factor (SCF), a key factor for mast cell survival [65]. Indeed, corticosteroid is a cornerstone of treatment for asthma exacerbations and dexamethasone has been shown to rapidly relieve symptoms, significantly reduce airway inflammation, and abrogate AHR [66–68]. The prophylactic administration in this study may also suggest that the regular use of inhaled corticoids may dampen the viral stimulation to cause exacerbations. However, in contrast to the marked effect on the pulmonary functional and inflammatory changes, we observed that dexamethasone did not affect the changes in IL-5, IL6 and IFNγ mRNA expression induced by the combination of the TLR3 and TLR7 agonists, indicating that these alterations neither influenced the pulmonary functional output nor the increase in lung inflammation and mast cells.

## Conclusion

This study showed that TLR agonists stimulation can mimic microbial infections, causing several asthma-like features, such as airway inflammation, AHR and increased mast cell numbers in guinea pigs. It is noteworthy that we found TLR agonists can induce an increase in mast cells. Their activation can release various mediators, such as histamine and leukotrienes, leading to airway constriction. The present results highlight that the role of mast cells in this process, as well as in viral infections, warrants further investigation in the future. Additionally, although TLR agonists were used to simulate an infection and could directly induce alterations in Penh, the sudden administration of a specific dose of TLR agonists differ from the gradual growth of the microbes in the airways. The relationship between TLR stimulation and natural occasion requires further investigation. However, the use of both TLR3 and TLR7 agonists is relevant when studying the effect of RV and RSV as real viruses sometimes are difficult to obtain and use. Moreover, dexamethasone has a therapeutic effect on abnormalities caused by TLR activations. Therefore, in the future, it is promising to use guinea pig and TLR agonists to investigate mechanism underlying viral-induced asthma exacerbations.

## Electronic supplementary material

Below is the link to the electronic supplementary material.

Supplementary Material 1

## Data Availability

No datasets were generated or analysed during the current study.

## Abbreviations

AHR: Airway Hyperresponsiveness
BALF: Bronchoalveolar Lavage Fluid
BECs: Bronchial Epithelial Cells
CXCL8: C-X-C Motif Chemokine Ligand 8
dsRNA: Double-Stranded RNA
IFNγ: Interferon Gamma
IL: Interleukin
LPS: Lipopolysaccharide
MCs: Mast Cells
PCLS: Precision-Cut Lung Slices
PBS: Phosphate-Buffered Saline
Penh: Enhanced Pause
PRRs: Pattern Recognition Receptors
RSV: Respiratory Syncytial Virus
RV: Rhinovirus
SCF: Stem Cell Factor
SEM: Standard Error of the Mean
ssRNA: Single-Stranded RNA
Th: T-helper (cells)
TLR: Toll-Like Receptor
