# Supplementary material for: Toll-like receptor activation induces airway obstruction and hyperresponsiveness in guinea pigs
Source: Respir Res. 2024 Nov 29;25:421. doi: 10.1186/s12931-024-03050-3 (PMC11607803; doi:10.1186/s12931-024-03050-3)
Supplement: Supplementary file 1 — Supplementary Material 1 [file 12931_2024_3050_MOESM1_ESM.pdf]

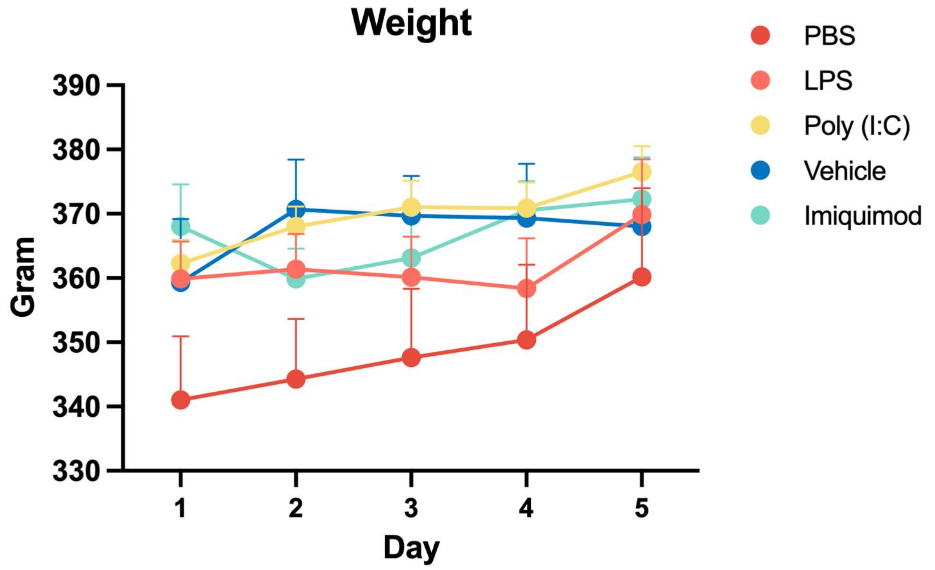

Supplementary Figure 1 The weight change curve of guinea pigs under different treatment.

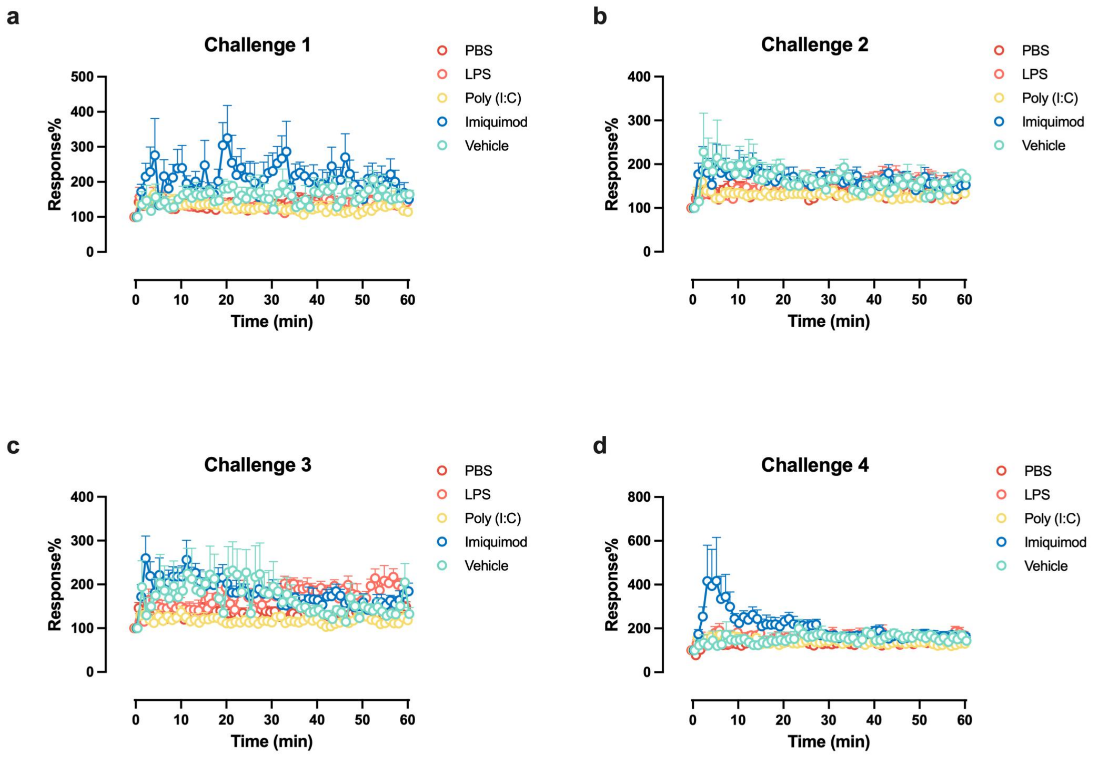

Supplementary Figure 2 Continuous changes in normalized Penh values (response%) over 60 minutes after intranasal administration with PBS, vehicle or TLR agonists.

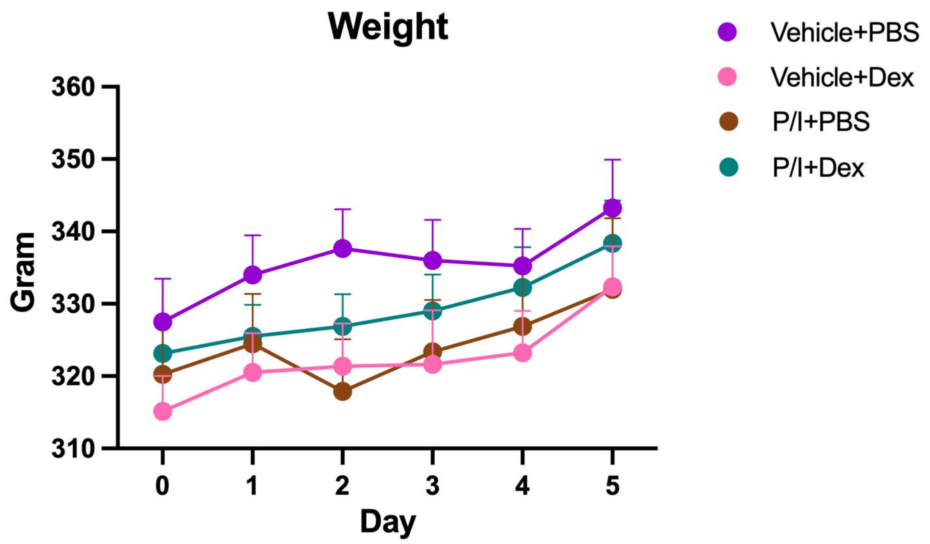

Supplementary Figure 3 The weight change curves of guinea pigs under different treatment combinations.

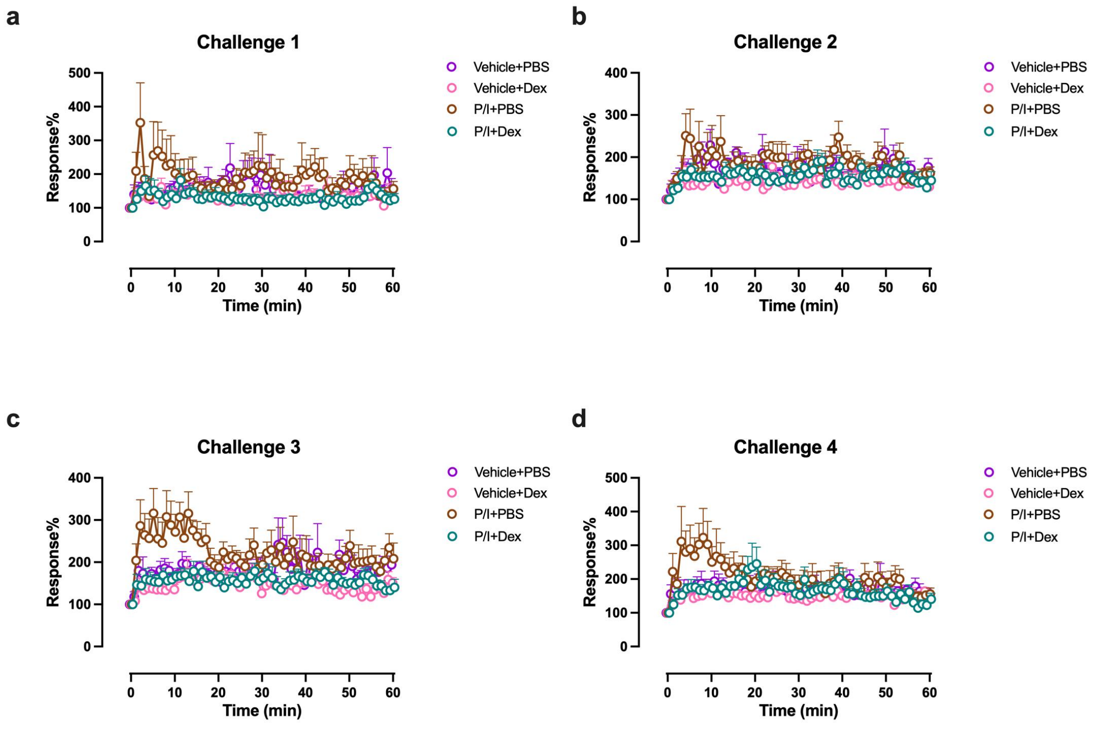

Supplementary Figure 4 Continuous changes in normalized Penh values (response%) over 60 minutes after intranasal administration with vehicle or P/I.
